# Health system determinants of infant, child and maternal mortality: A cross-sectional study of UN member countries

**DOI:** 10.1186/1744-8603-7-42

**Published:** 2011-10-24

**Authors:** Katherine A Muldoon, Lindsay P Galway, Maya Nakajima, Steve Kanters, Robert S Hogg, Eran Bendavid, Edward J Mills

**Affiliations:** 1 School of Population and Public Health, University of British Columbia, 2206 East Mall, Vancouver, British Columbia, Canada; 2 British Columbia Centre for Excellence in HIV/AIDS, St. Paul's Hospital, 1081 Burrard Street, Vancouver, British Columbia, Canada; 3 Faculty of Health Sciences, Simon Fraser University, 888 University Drive, Burnaby, British Columbia, Canada; 4 Division of General Internal Medicine, Stanford University, Palo Alto, California, USA; 5 Faculty of Health Sciences, University of Ottawa, Roger Guindon Hall 451, Smyth Road, Ottawa, Ontario, Canada

## Abstract

**Objective:**

Few studies have examined the link between health system strength and important public health outcomes across nations. We examined the association between health system indicators and mortality rates.

**Methods:**

We used mixed effects linear regression models to investigate the strength of association between outcome and explanatory variables, while accounting for geographic clustering of countries. We modelled infant mortality rate (IMR), child mortality rate (CMR), and maternal mortality rate (MMR) using 13 explanatory variables as outlined by the World Health Organization.

**Results:**

Significant protective health system determinants related to IMR included higher physician density (adjusted rate ratio [aRR] 0.81; 95% Confidence Interval [CI] 0.71-0.91), higher sustainable access to water and sanitation (aRR 0.85; 95% CI 0.78-0.93), and having a less corrupt government (aRR 0.57; 95% CI 0.40-0.80). Out-of-pocket expenditures on health (aRR 1.29; 95% CI 1.03-1.62) were a risk factor. The same four variables were significantly related to CMR after controlling for other variables. Protective determinants of MMR included access to water and sanitation (aRR 0.88; 95% CI 0.82-0.94), having a less corrupt government (aRR 0.49; 95%; CI 0.36-0.66), and higher total expenditures on health per capita (aRR 0.84; 95% CI 0.77-0.92). Higher fertility rates (aRR 2.85; 95% CI: 2.02-4.00) were found to be a significant risk factor for MMR.

**Conclusion:**

Several key measures of a health system predict mortality in infants, children, and maternal mortality rates at the national level. Improving access to water and sanitation and reducing corruption within the health sector should become priorities.

## Background

A working definition of a health system, as proposed by the World Health Organization (WHO) is a system "whose primary purpose is to promote, restore, or maintain health" [1]. In 2007, with the purpose of promoting a common understanding of what a health system is and action areas for strengthening health systems, the WHO developed a framework composed of six building blocks of a health system: 1) health service coverage, 2) human health resources, 3) health information systems, 4) medical products, vaccines and technology, 5) health financing, and 6) leadership and governance [2]. These building blocks aim to support a health system that can prevent, treat and manage illness and to preserve mental and physical well-being for all individuals equitably and efficiently, within a specified geographic area. Health system activities range from direct service provision through clinics and hospitals to community level prevention strategies and health education. Over the past decade there has been renewed interest in the horizontal role of health systems in the promotion and maintenance of health [3]. Additionally, the robustness of a public health system has been highlighted as a necessary component to achieve the Millennium Development Goals (MDG) [4,5], however the indicators to measure health system strengthening are less understood.

There is an on-going debate about global health 'geometry' of the vertical or horizontal approaches to health as both have strengths and limitations [6-8]. Both private and public systems can employ vertical and horizontal approaches to health care and programming and some have even used the term 'diagonal' to describe combining the two approaches to optimize processes and outcomes [9]. A notable trend is that private organizations tend to have a more narrow focus and employ a more vertical approach. For example, in many low-income countries (LIC), externally led, donor driven projects have met with some success, especially with the establishment of care centres for the treatment and prevention of HIV/AIDS, immunization coverage, TB control, and Roll Back Malaria Campaigns, all typically considered a vertical approach to health. These disease-focused initiatives are intensive, may avoid the bureaucracies and inefficiencies of a national health system, and are typically implemented to either respond to an emergency (as in the case of HIV/AIDS) or meet donor specific requirements (such as vaccines through GAVI, the Global Alliance for Vaccines and Immunizations). However, investments aimed at the overall strength and functioning of a health system (i.e. horizontal approaches to health) are grounded in the expectation that a functioning, efficient health care system will contribute most effectively to improving the health of a population [10].

Although some countries have made substantial improvements in infant, child and maternal mortality rates (IMR, CMR, MMR respectively) during the last century, improvements have slowed and even reversed in some nations during the last few decades [11]. An estimated 9.7 million children under-five die worldwide each year [12]. Additionally, mortality rates are highly variable across nations highlighting health inequities and larger social and environment determinants that predispose some nations to higher rates of mortality [13]. Differences in all-cause mortality rates across nations may, in part, be explained by the strength and functioning of a national health system's ability to safe-guard health beyond the disease specific approach. Important funding agencies such as the US Global Health Initiative, are now directing their financial contributions to health system strengthening at the expense of disease focused initiatives, even though validated indicators to determine and monitor health systems strength are not well determined or understood [14]. We aimed to develop an exploratory analysis to examine the strength of association between important public health endpoints (IMR, CMR, MMR) and potential indicators of health system strength and functioning as theorized by the WHO using publicly available data.

## Methods

### Data and variables

Variable selection was informed *a priori *by the WHO building block framework. The goal was to select variables that could represent each of the 6 building blocks and then to investigate how well they explain the variability in global mortality rates. All data was publicly access so variable selection was constrained by data availability. Data on ten indicators categorized into five of the six main building blocks of a health system as outlined by the WHO, and four relevant demographic variables were used as explanatory variables. Nursing and midwife density and physician density measured available human health resources. Vaccines coverage was indicated by the percentage of children receiving measles immunizations annually. Health service delivery was represented by the percentage of the population with sustainable access to water and sanitation and the percentage of births attended by skilled attendants. Health financing was assessed by total, out-of-pocket, government, and private expenditures on health. The health finance data was gathered from WHOSIS. They cite that all financial measurements are made using the "International dollar rate [which] is a common currency unit that takes into account differences in relative purchasing power annual average".

Finally, The Corruption Perception Index, a metric designed to measure the perceived levels of public sector corruption published annually by Transparency International, was used to measure the governance and leadership category [15]. Although the CPI focuses on perceptions of corruption rather than the actual extent of corruption, the index has been assessed to be a reliable and consistent measure [16]. The final building block of a health system is health information systems that can be captured by the presence of a functioning surveillance system, however multinational data was not available for this building block. Together these indicators act as a proxy representing the robustness of national health systems to finance, staff, and provide health services to their citizens. Demographic variables included fertility rate, national population growth, urban population growth, and female labour force participation and were used to capture demographic heterogeneity across countries.

We extracted all data from our prospectively maintained archive of publicly accessible health statistics, named the Globally Accumulated health Indicator Archive (GAIA). Source data for the outcome and explanatory variables originated from UN and WHO data, with the exception of the CPI, which originated from Transparency International; all publicly available sources. The outcome variables are based on 2008 data while the explanatory variables were collected over a seven-year span from 2001-2008 using the most recent data available. Of 192 UN member countries, 136 countries provided sufficient data for the chosen variables. Eight of the 136 countries would have been excluded due to lack of data on sustainable access to water. Rather than excluding these countries, we assumed 95% value for Poland and Portugal and assumed 100% for Belgium, France, Ireland, Italy, New Zealand, and the United Kingdom (the median value for Australia, and Western European and North American countries). Without this assumption the countries from Western and Southern Europe were under-represented.

### Statistical Analysis

Descriptive statistics were used to display the dispersion of the outcome and explanatory variables. A linear mixed effect model was chosen to account for the natural geographic clustering of the countries according to UN sub-region classification. In order to comply with the strict conditions of linear modeling, some transformations were required. Each outcome required a logarithmic transformation. Nursing and midwife density, total government spending, out-of- pocket expenditures, government expenditures and fertility rate were transformed via logarithm. Measles immunization and skilled birth attendants were dichotomized as 90% or more and under 90% based on the scatter plot indicating a clear drop-off after 90%.

Multicollinearity was an issue as the variance inflation factors (VIF) was high for government health expenditures. Upon removing government expenditures, the VIF were moderate in size, reaching a maximum value of 6.21 when considering the full model prior to model selection. Model conditions were assessed through analysis of marginal and conditional residuals. Model selection was achieved by minimizing the Akaike Information Criterion (AIC) while keeping all type III p-values for covariates below 0.20. Unadjusted results consider the association between the outcome and each explanatory variable individually. Adjusted risk ratios consider the association between the outcome and an explanatory variable simultaneous to all variables selected in the model. Variables selected in the multivariate models are considered the strongest predictors because the non-selected variables are no longer informative with respect to the outcome. All analyses were done by SK using SAS 9.1.3 [17].

Ethics approval for this project was not required because it uses publicly available data.

## Results

**Figure 1:**
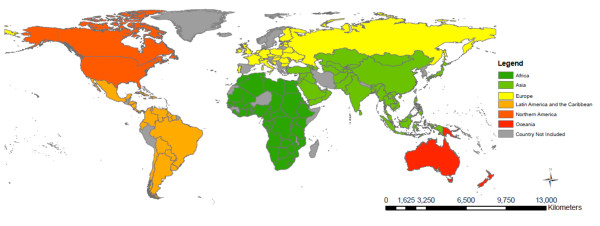
**Countries included in analysis (n = 136) **.

**Figure 2:**
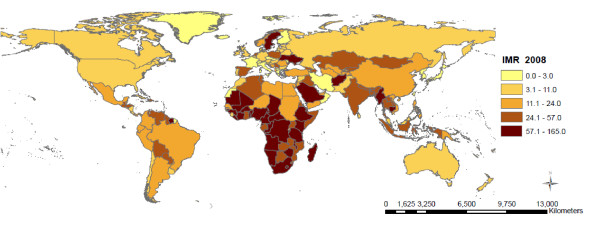
**Infant mortality rate per 1000 live births across countries (n = 136) **.

**Figure 3:**
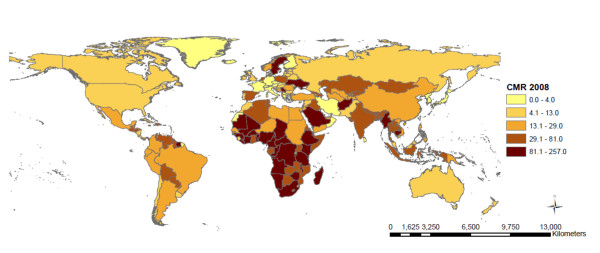
**Child mortality rate per 1000 live births across countries (n = 136) **.

**Figure 4:**
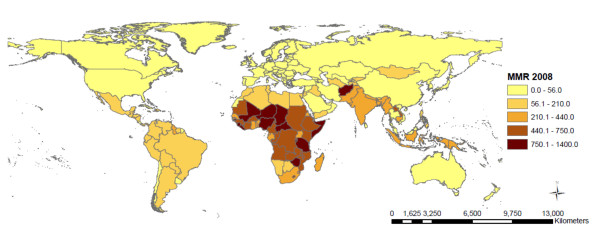
**Maternal mortality rate per 100,000 live births across countries (n = 136) **.

The descriptive statistics for each of the outcome measures (IMR, CMR, MMR) and the explanatory variables are included in Table 1. The median IMR across all nations was 21.5 deaths per 1,000 live births (IQR 10.0 - 60.0), median CMR was 24.5 deaths per 1,000 live births (IQR 11.0-80.0) and median MMR was 81.5 deaths per 100,000 live births (IQR 26.0-350.0). The geographic classification of the 136 countries included in this study is shown in Table 2. Of the 136 countries, 46 (33.8%) of the countries are located in Sub-Saharan Africa; 39 (28.7%) in Asia; 25 (18.4%) in Europe; 21 (15.4%) in Latin America and the Caribbean; 2 (1.8%) in North America; and 3 (2.2%) in Oceania. The proportion of countries included in the model varies between regions, where over 80% of all Sub-Saharan countries are included but only 12% of Oceanic countries had sufficient data available for inclusion in this model. The countries included in the analysis and the mortality rates are represented in Figure 1, Figures 2, 3, and 4 show the global distribution of mortality rates in 2008.

**Table 1:** Descriptive statistics for all outcome and explanatory variables sub-divided into the WHO framework for the building blocks of a health system (n = 136 countries)

Variables	Median (IQR)	Range
**Outcome**		
Infant mortality rate (per 1,000 births)	21.5 (10.0 - 60.0)	2.0 - 165.0
Child mortality rate (per 1,000 births)	24.5 (11.0 - 80.0)	3.0 - 257.0
Maternal mortality ratio (per 100,000 births)	81.5 (26.0 - 350.0)	3.0 - 1400.0
**Explanatory**		
**I. Human health resources**		
Nursing/midwife density (per 10,000 population)	18.5 (7.0 - 51.0)	2.0 - 158.0
Physician density (per 1,000 population)	11.0 (2.0 - 25.0)	0.3 - 64.0
**II. Health service coverage**		
% Of population with sustainable access to water and sanitation	87.50 (59.0 - 98.5)	24.0 - 100.0
% Of births attended by skilled staff	93.0 (57.0 - 100.0)	6.0 - 100.0
**III. Medical products, vaccines and technology**		
% Measles immunization coverage	91.0 (79.0 - 97.0)	23.0 - 99.0
**IV. Health financing**		
Total health expenditure per person (USD)	153.0 (35.5 - 441.0)	4.0 - 6714.0
Out-of-pocket expenditure on health (as a % of total health expenditure)	33.1 (19.8 - 48.4)	4.2 - 82.7
Government health expenditure (USD)	148.0 (41.0 - 457.5)	4.0 - 3074.0
Private share of total health expenditure (%)	44.8 (27.9 - 58.5)	9.3 - 83.6
**V. Leadership and governance**		
Corruption Index	3.0 (2.4 - 4.5)	1.3 - 9.4
**Demographic variables**		
Fertility rate (average number of children per woman)	2.5 (1.8 - 4.1)	1.2 - 6.6
Population growth value (annual %)	1.42 (0.72 - 2.29)	-1.17 - 5.32
Urban population value (annual %)	2.23 (1.16 - 3.35)	-1.02 - 5.90
Female labour force participation (%)	59.8 (48.5 - 68.1)	14.9 - 90.2

Lower value of Corruption Index on a scale of ten indicates higher perceived corruption

**Table 2:** Descriptive classification of the study countries (n = 136 countries)

Region	N (%)	Total number of countries by region, % included in the analysis by region
Africa	46 (33.8)	57 (80.7)
Asia	39 (28.7)	50 (78.0)
Europe	25 (18.4)	51 (49.0)
Latin America and the Caribbean	21 (15.4)	48 (43.8)
North America	2 (1.5)	5 (40.0)
Oceania	3 (2.2)	25 (12.0)
Total	136 (100.0)	236

All selected health system indicators were significantly associated with IMR at the bivariate level except for population growth and female labour force participation, and were therefore included in the multiple regression analysis. When controlling for the effects of other variables in the model, four variables remained significantly associated with IMR. Health system determinants associated with lower IMR are higher physician density (adjusted rate ratio [aRR] 0.81; 95% CI 0.71-0.91), higher sustainable access to water and sanitation (aRR 0.85; 95% CI 0.78-0.93), and having a less corrupt government (aRR 0.57; 95% CI 0.40-0.80). Out-of-pocket expenditure on health (a-RR 1.29; 95% CI 1.03-1.62) was associated with higher for IMR (see Table 3).

**Table 3:** Linear mixed effect regression analysis results for IMR, 2008 sub-divided into the WHO framework for the building blocks of a health system (n = 136 countries)

Explanatory Variables	Unadjusted Risk Ratio (95% CI)	Adjusted Risk Ratio (95%CI)
**I. Human health resources**		
Nursing/midwife density (per 10,000 population)	0.82 (0.71, 0.94)	-
Physician density (per 1,000 population)	0.72 (0.63, 0.83)	0.81 (0.71, 0.91)
**II. Health service coverage**		
% Of population with sustainable access to water and sanitation (for a 10% increase)	0.74 (0.68, 0.80)	0.85 (0.78, 0.93)
% Of births attended by skilled staff	0.28 (0.20, 0.39)	-
**III. Medical products, vaccines and technology**		
% Measles immunization coverage	0.71 (0.52, 0.98)	-
**IV. Health financing**		
Total health expenditure per person (USD)	0.74 (0.67, 0.82)	-
Out-of-pocket expenditure on health (as a % of total health expenditure)	1.60 (1.28, 2.01)	1.29 (1.03, 1.62)
Government health expenditure (USD)	0.65 (0.58, 0.71)	-
Private share of total health expenditure (%)	1.01 (1.00, 1.02)	-
**V. Leadership and governance**		
Corruption index (log of)	0.37 (0.26, 0.53)	0.57 (0.40, 0.80)
**Demographic variables**		
Fertility rate (average number of children per woman)	3.07 (2.04, 4.62)	-
Population growth value (annual %)	1.20 (1.01, 1.43)	-
Urban population value (annual %)	1.26 (1.12, 1.43)	-
Female labour force participation (%)	1.00 (0.99, 1.01)	-

- : Not selected in final model

CI: Confidence interval

A Risk Ratio below 1 corresponds to a protective variable

A Risk Ratio above 1 corresponds to a risk factor

The same four variables that were significantly associated with IMR were also significant for CMR after controlling for other factors (see Table 4). Higher physician density (aRR 0.80; 95% CI 0.70-0.92), higher sustainable access to water and sanitation (aRR 0.82, 95% CI 0.75-0.91), and having a less corrupt government (a-RR 0.58; 95% CI 0.40-0.84) were associated with lower CMR. Out-of-pocket expenditures on health (aRR 1.29; 95% CI 1.01, 1.65) was significantly associated with higher CMR.

**Table 4:** Linear mixed effect regression analysis results for CMR, 2008 sub-divided into the WHO framework for the building blocks of a health system (n = 136 countries)

Explanatory Variables	Unadjusted Risk Ratio (95% CI)	Adjusted Risk Ratio (95%CI)
**I. Human health resources**		
Nursing/midwife density (per 10,000 population)	0.80 (0.69, 0.93)	-
Physician density (per 10,000 population)	0.71 (0.61, 0.82)	0.80 (0.70, 0.92)
**II. Health service coverage**		
% Of population with sustainable access to water and sanitation (for a 10% increase)	0.71 (0.65, 0.77)	0.82 (0.75, 0.91)
% Of births attended by skilled staff	0.48 (0.32, 0.72)	-
**III. Medical products, vaccines and technology**		
% Measles immunization coverage	0.67 (0.48, 0.94)	-
**IV. Health financing**		
Total health expenditure per person (USD)	0.73 (0.66, 0.82)	-
Out-of-pocket expenditure on health (as a % of total health expenditure)	1.64 (1.28, 2.10)	1.29 (1.01, 1.65)
Government health expenditure (USD)	0.63 (0.56, 0.70)	-
Private share of total health expenditure (%)	1.01 (1.00, 1.02)	-
**V. Leadership and governance**		
Corruption index ( log of)	0.35 (0.24, 0.52)	0.58 (0.40, 0.84)
**Demographic variables**		
Fertility rate (average number of children per woman)	3.54 (2.28, 5.49)	-
Population growth value (annual %)	1.25 (1.04, 1.52)	-
Urban population value (annual %)	1.31 (1.15, 1.50)	-
Female labour force participation (%)	1.00 (0.99, 1.01)	-

- : Not selected in final model

CI: Confidence interval

A Risk Ratio below 1 corresponds to a protective variable

A Risk Ratio above 1 corresponds to a risk factor

Finally, higher sustainable access to water and sanitation (aRR 0.88; 95% CI 0.82-0.94), having a less corrupt government (aRR 0.49; 95% CI 0.36-0.66), and higher total expenditures on health per capita (a-RR 0.84; 95% CI 0.77-0.92) were associated with lower MMR. It should be noted that higher fertility rate (aRR 2.85; 95% CI 2.02-4.00) is a significant risk factor for MMR (see Table 5).

**Table 5:** Linear mixed effect regression analysis results for MMR, 2008 sub-divided into the WHO framework for the building blocks of a health system (n = 136 countries)

Explanatory Variables	Unadjusted Risk Ratio (95% CI)	Adjusted Risk Ratio (95%CI)
**I. Human health resources**		
Nursing/midwife density (per 10,000 population)	0.76 (0.66, 0.87)	-
Physician density (per 1,000 population)	0.68 (0.58, 0.79)	-
**II. Health service coverage**		
% Of population with sustainable access to water and sanitation (for a 10% increase)	0.67 (0.61, 0.73)	0.88 (0.82, 0.94)
% Of births attended by skilled staff	0.28 (0.20, 0.39)	-
**III. Medical products, vaccines and technology**		
% Measles immunization coverage	0.55 (0.40, 0.74)	-
**IV. Health financing**		
Total health expenditure per person (USD)	0.60 (0.55, 0.65)	0.84 (0.77, 0.92)
Out-of-pocket expenditure on health (as a % of total health expenditure)	1.32 (1.04, 1.66)	-
Government health expenditure (USD)	0.53 (0.48, 0.58)	-
Private share of total health expenditure (%)	1.01 (1.00, 1.02)	-
**V. Leadership and governance**		
Corruption index (log of)	0.18 (0.13, 0.23)	0.49 (0.36, 0.66)
**Demographic variables**		
Fertility rate (average number of children per woman)	9.93 (6.96, 14.16)	2.85 (2.02, 4.00)
Population growth value (annual %)	1.07 (0.89, 1.28)	-
Urban population value (annual %)	1.33 (1.17, 1.51)	-
Female labour force participation (%)	1.00 (0.99, 1.02)	-

- : Not selected in final model

CI: Confidence interval

A Risk Ratio below 1 corresponds to a protective variable.

A Risk Ratio above 1 corresponds to a risk factor

### Interpretation

This ecological analysis explores how the WHO building blocks of a health system are associated with infant, child and maternal mortality rates across 136 UN member countries. Service coverage as measured by sustainable access to water is associated with decreased mortality. Leadership and governance as measured by the corruption index (i.e. less government corruption) are associated with decreased mortality. Human health resources as measured by physician density, and health financing as measured by less out-of-pocket payments are associated with decreased mortality but only for infants and children.

Stewardship is a neglected function in most health systems [18]. Murray & Frenck (2000) have described health system stewardship as involving three key aspects "setting, implementing and monitoring the rules for the health system; assuring a level playing field for all actors in the system; and defining strategic directors for the health system as a whole". Currently there is no one metric to measure health stewardship at the national level, we used the Corruption Index as a measure of national governance and a proxy for health system stewardship because the general functioning of the government can strongly influence stewardship and regulation. Corruption is broadly defined by Transparency International as the misuse of public office for private gain [19]. As a result, our findings are limited to corruption within the public sphere although we do acknowledge that corruption is present in the private and non-governmental arena. In our study we have found that the more corrupt a government is perceived to be (i.e. lower CPI score) the stronger the association with increased rates of infant, child and maternal mortality.

As health systems are publicly administered and require strong national commitment and resources, a corrupt government runs the risk of diverting public health resources for private gains. Our findings suggest that transparent governance is an essential component of health system strengthening and an important pathway to improve population health. Three quarters of the countries in the world have a CPI score less than five, translating to a serious level of corruption [20], as a result it has been recognized by the UN that anti-corruption should be a central approach to global aid and development [21]. Corruption is systemic and exists within and across scales and sectors of the government and thus requires anti-corruption efforts that are both broad and sector-specific. Private vertical programs are often fast and effective because they often operate outside the public sphere, however an unintended consequence of this approach could be enabling a cycle of corruption within the public sphere. Public health exists and is implemented within the larger public system, and therefore must incorporate wherever possible policies that buttress transparency among participating stakeholders from multiple disciplines [22].

Sustainable access to water and sanitation was significantly associated with IMR, CMR and MMR when controlling for other variables presumably for several reasons. Elevated incidence and prevalence rates of diarrhoeal diseases are commonly observed in settings with limited access to improved and sustainable water and sanitation services. Foreign aid is associated with increased access to water, but not necessarily sanitation [23]. Water-borne diarrhoeal diseases alone account for 17% of deaths in children under-five and 1% of neonatal deaths [12]. Other ecological level studies have also shown that MMR is strongly associated with sustainable access to water and sanitation because access to safe drinking water is a fundamental pillar for maternal health [24]. Unhygienic birthing practices and facilities that are not properly equipped to provide a sterile environment for a post-partum mother commonly contribute to elevated rates of maternal mortality. Mothers who are unable to breast-feed are at risk of using unsafe water for formula-feeding especially in low income countries as a mode of prevention of mother-to-child transmission of HIV [25].

Health financing was a central finding across all three models. Each financial variable with the exception of private share of total health expenditure was significantly related to mortality outcomes, but once we included them within the multivariate model out-of-pocket best explained IMR and CMR, and total health expenditure best describes the MMR. This finding is not indicative that out-of-pocket is not important for MMR, or that total health expenditure is not important for IMR and CMR, but rather that the model selected the variable that described the strongest association. Out-of-pocket expenditure is a commonly cited barrier to health care especially if out-of-pocket costs exceed household income. In many African countries, the health financing system is too weak to function without the cushion of out-of-pocket costs. In a study of 15 African countries investigating household coping behaviours in the face of health expenditures, it was found that between 23-68% of households would resort to borrowing and selling their assets [26]. Households in this situation are often affected by both the cost of medical care, but also the loss of income from sick family members that cannot work [26]. This contributes to a highly inequitable system that puts infants and children at increased risk for adverse health outcomes and death.

Although we cannot tell the temporality of this relationship, we observe that as per capita spending on health increases mortality rates decrease. Others have shown that total health expenditures is a significant predictor of IMR in their bivariate analysis, however, this is no longer significant in the multivariate model, after including Gross National Income per capita [11]. This was the same for our analysis and probably points to the larger influence of a countries economic status (i.e. GNI) rather than the amount of funding earmarked for health care.

Physician density significantly reduces infant and child mortality but does not appear to reduce maternal mortality after controlling for other health system indicators, nor does nursing and midwife density. There have been at least six cross-national studies that have investigated human health resources, indicated by either physician or nurse densities as predictors of infant mortality [4,27-32]. Of these studies, four found no relationship between human health resources while two of the more recent studies have indicated that both physician and nurse densities are significant in accounting for variations in rates of infant mortality across countries. Interestingly, Farahani *et al*. (2009) have shown, using longitudinal panel data to examine both the short- and long-term effects of human health resources, that human health resources may have greater long-term benefits than previously estimated. We chose not to use an amalgamated measure (i.e. nurses, doctors, skilled birth attendants) for human health resources and found that physician density was significant yet nurse density and % of births with a skilled attendant was not significant. This could be due to the fact that some countries only include professional nurses while associate profession such as nursing assistants are not included [33]. This would under-represent the role that nurses play in human health resources.

The MDG #6 was designed to improve maternal health because it is estimated that in some areas of the world a woman has a 1 in 16 chance of dying in pregnancy. High infant, child and maternal mortality are often observed concurrently with high fertility, however only MMR was positively and significantly associated with fertility in our analysis. It is widely supported that a high fertility rate is observed in settings where children are not surviving and families need to replace the lost children. If a woman has had a complication during a previous pregnancy or her health becomes compromised this can lead to a vicious circle that puts mothers (and children) at risk for malnourishment and health complications [34].

In 1990, The World Summit for Children called for a reduction in infant mortality to below 70 deaths per 1000 live births (or a one third reduction if this resulted in a lower mortality rate) by the year 2000 [12,35]. This goal was attained by discouragingly few nations; a failure that some suggest may be rooted in inadequate investments in health and limited improvements in the strength and functioning of health systems [35]. Results from our analyses show that more up-stream determinants such as sustainable access to water and sanitation, health financing, and transparent governance are important pathways to reducing mortality rates. Health financing is not currently listed within the MDGs however the latest WHO report [36] focuses exclusively on sustained economic and social development to move towards universal coverage and improved health outcomes. Studies such as this are needed to strengthen our current understanding of the role of health systems as a societal safety net in achieving the MDGs and improving health worldwide.

## Limitations

Several limitations should be considered when interpreting these results. Data selection was constrained primarily by data availability and therefore does not include the most comprehensive list of health system indicators. Our sample size (n = 136 countries) also constrains our choices for the number of variables that we can include in the model. As a result we have a relatively small number of variables used to describe the variability and complex nature of a health system. This study is a cross-sectional analysis at the country level and therefore we cannot draw causal inferences from the results. As we have used countries as the unit of analysis, this does not provide any information about variation within the nation state. This is an important point to stress because health status throughout a country may vary tremendously and these differences will be masked by country-level data. While many studies have controlled for female education as an important variable related to infant mortality, we did not include this as an explanatory variable because the data was not adequately populated [11]. In place, we used the indicator for female labour involvement. While our study focused on outcomes of maternal and child health we recognize that men are one of the highest risk groups for early mortality, yet are not the focus of any large directed funding initiatives, with the possible exception of male circumcision [37].

## Conclusion

In conclusion, our analysis identifies the importance of several key indicators of health system strength and functioning that are significantly associated with infant, child and maternal survival at the national aggregate level and after controlling for other health system determinants and demographic factors. The strength of a health system offers an important and sustainable mechanism to influence key population level indicators of health. There is now an important need to understand indicators of health system strength at the local level and how to improve health system strength and functioning in practice.

## Competing interests

The authors declare that they have no competing interests.

## Ethics

Ethics approval for this project was not required because it uses publicly available data.

## Authors' contributions

KAM, LPG and MN contributed equally to the drafting, interpretation and incorporation of critical feedback from co-authors. SK conducted the statistical analysis and assisted with interpretation. EB, RSH and EJM supervised, drafted, and provided critical feedback at all stages of the manuscript. All authors read and approved the final manuscript.
